# The Evaluation of a Social Media Campaign to Increase COVID-19 Testing in Migrant Groups: Cluster Randomized Trial

**DOI:** 10.2196/34544

**Published:** 2022-03-24

**Authors:** Ingeborg Hess Elgersma, Atle Fretheim, Thor Indseth, Anita Thorolvsen Munch, Live Bøe Johannessen, Christine Engh Hansen

**Affiliations:** 1 Centre for Epidemic Interventions Research Norwegian Institute of Public Health Oslo Norway; 2 Faculty of Health Sciences Oslo Metropolitan University Oslo Norway; 3 Cluster for Health Services Research Norwegian Institute of Public Health Oslo Norway; 4 Division of Prevention and Public Health Norwegian Directorate of Health Oslo Norway; 5 Mindshare Norway Oslo Norway

**Keywords:** COVID-19, SARS-CoV-2, social media, campaign, cluster randomized trial, nonpharmaceutical interventions, migrant, intervention, testing, strategy, public health, Facebook, communication

## Abstract

**Background:**

A low test positivity rate is key to keeping the COVID-19 pandemic under control. Throughout the pandemic, several migrant groups in Norway have seen higher rates of confirmed COVID-19 and related hospitalizations, while test positivity has remained high in the same groups. The Norwegian government has used several platforms for communication, and targeted social media advertisements have in particular been an important part of the communication strategy to reach these groups.

**Objective:**

In this study, we aimed to investigate whether such a targeted Facebook campaign increased the rate of COVID-19 tests performed in certain migrant groups.

**Methods:**

We randomly assigned 386 Norwegian municipalities and city districts to intervention or control groups. Individuals born in Eritrea, Iraq, Pakistan, Poland, Russia, Somalia, Syria, and Turkey residing in intervention areas were targeted with a social media campaign aiming at increasing the COVID-19 test rate. The campaign message was in a simple language and conveyed in the users’ main language or in English.

**Results:**

During the 2-week follow-up period, the predicted probability of having a COVID-19 test taken was 4.82% (95% CI 4.47%-5.18%) in the control group, and 5.58% (95% CI 5.20%-5.99%) in the intervention group (*P*=.004).

**Conclusions:**

Our targeted social media intervention led to a modest increase in test rates among certain migrant groups in Norway.

**Trial Registration:**

ClinicalTrials.gov NCT04866589; https://clinicaltrials.gov/ct2/show/NCT04866589

## Introduction

Several migrant groups have been disproportionally affected by COVID-19 in Norway [1,2]. These groups are also considered difficult to reach through traditional communication platforms [3,4]. Adapting, translating, and targeting communication to ensure equal access to information regardless of health literacy and language skills, is an important part of crisis communication. Consequently, Norwegian health authorities have used a wide range of platforms to communicate with these groups during the pandemic: public COVID-19 websites, traditional media outlets such as television, radio, boards and printed advertisements, as well as content marketing, contextual digital ads, targeted cell phone messaging, influencers, and a wide array of social media such as Twitter, Instagram, Snapchat, TikTok, YouTube, and Facebook. Certain migrant groups have seen particularly high COVID-19 prevalence and low test rates [5], and social media campaigns can be crucial to reach these groups. Although such campaigns have been monitored in house in terms of outreach and reactions on the social media platform, their effects on behavioral change have not been assessed.

Testing is key to detecting cases and limiting outbreaks. In this study, we aimed at assessing whether a targeted social media advertising campaign encouraging selected migrant groups to get tested affected testing behavior (ClinicalTrials.gov NCT04866589; protocol is provided in Multimedia Appendices 1 and 2). The intervention described in this paper was an extension of the “Get tested” campaign, which had been launched after the Norwegian Institute of Public Health (NIPH) published findings that indicated higher rates of undetected cases among certain migrant groups compared to the general population; that is, lower test rates, higher test positivity rates, and higher rates of hospitalization [1]. The “Get tested” campaign was a part of a larger effort directed at the migrant population, which included drop-in testing stations [6] and dialogue meetings with migrant groups.

The campaign was launched by the end of May 2021, when the COVID-19 incidence rate in Norway was decreasing, but the test positivity rate—that is, the share of all tests that are positive—remained high in many migrant groups [5]. Furthermore, there were indications of confusion concerning when to test and whether testing was free. In order to assess the campaign’s effect on the rates of testing, we ran a cluster randomized trial with users of Facebook. According to numbers from the Ipsos SoMe-tracker, 82% of the Norwegian population, aged ≥18 years, have a profile, and 69% of them are daily users on this social media platform [7]. The campaign targeted users residing in Norway, who were born in Eritrea, Iraq, Pakistan, Poland, Russia, Somalia, Syria, and Turkey. The groups were chosen because of their previous high test positivity levels. This study aimed to establish whether the targeted Facebook campaign increased the rate of testing in the selected migrant groups.

## Methods

### Trial Design

The trial was designed as a parallel group, 2-arm, superiority cluster randomized trial. The clusters—that is, municipalities and city districts—were allocated either to the intervention group or to the control group, where the intervention group received the campaign message and the control group did not receive the campaign message. All municipalities or city districts with at least one person in the target group were included in the study. Randomization was carried out at the cluster level as it was not technically possible to target the campaign to individual persons. The clusters were split evenly between treatments through block randomization with block size two. Before block randomization took place, the list of municipalities and city districts were sorted in accordance with the total number of migrants residing in each area. Partial concealment was ensured as team member 1 created and sorted the list, while team number 2 created a list of pairwise random orders of pairs of A and B, using randomization software [8]. Team number 3 combined these two lists.

### Intervention

Norwegian health authorities have, since the onset of the pandemic, carried out campaigns on social media directed toward the migrant population, using language-based and geographic targeting segments. In December 2020 a “Get tested” campaign was launched, aiming to increase the test rate among migrant populations. Prior to our trial, there were indications of an increased disinterest toward the message, as measured by reach and reactions, and the campaign was paused. To prevent message fatigue [9,10], campaign messages were continuously reviewed, performance measured and customized, and A/B testing conducted—this involved testing different colors, visuals, or creatives to determine which visual received more attention and the time spent on the message. As the “Get tested” campaign had been running for a while, there was by the end of May a small concern that the public had become disengaged or blind to the message; hence, it was decided to roll out new versions. The new versions used short, direct messaging on an A/B-tested colored background. The message remained the same.

**Figure 1:**
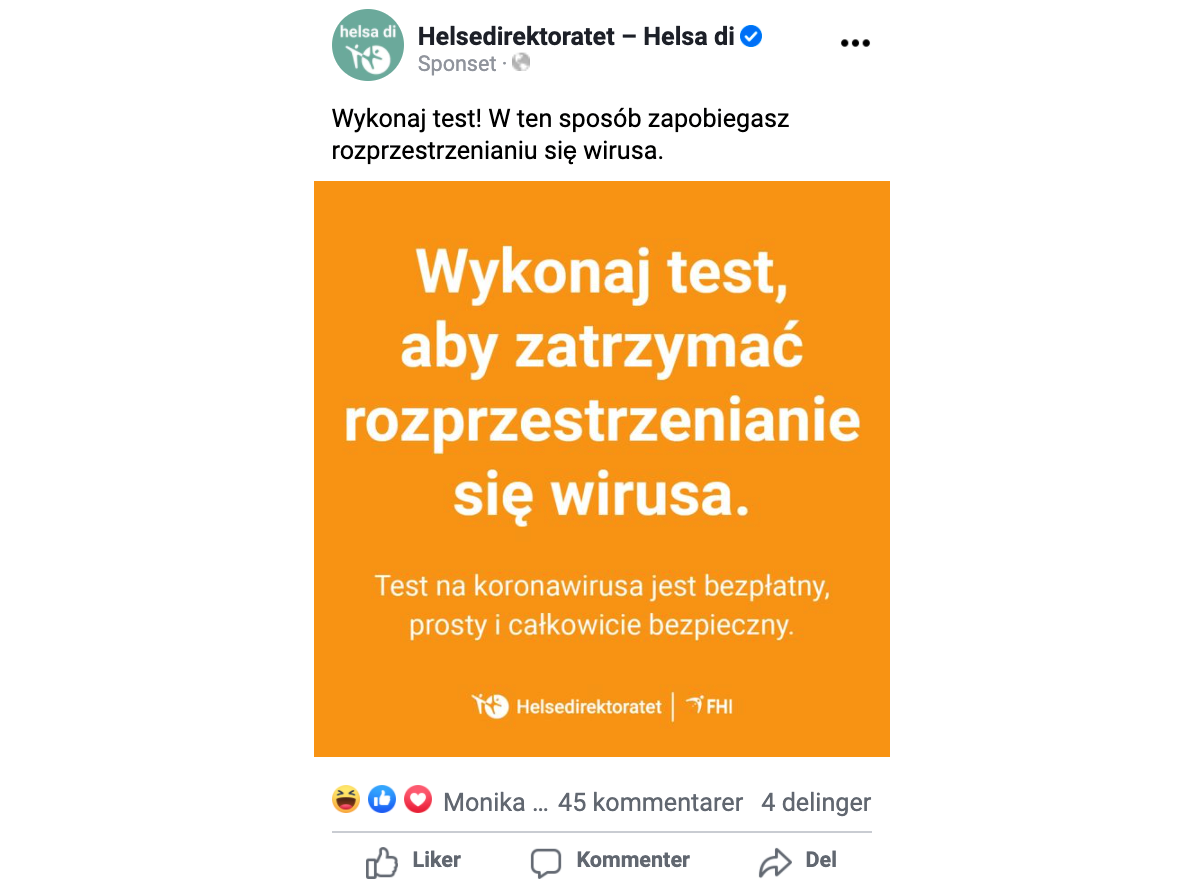
Screenshot of the social media post in Polish.

We decided that the distribution of the new campaign would be randomized by municipality or city district in order to facilitate an effect evaluation. The content of the relaunched campaign was a sponsored Facebook post from the Norwegian Directorate of Health, distributed in Tigrinya, Polish, Urdu, Somali, Russian, Kurmanji, English, Arabic, Turkish, and Sorani, and focused on the importance of testing for COVID-19 and that testing is free of charge and easy to take. The English message read the following: “COVID-19 tests are free, simple, and completely safe. Get yourself tested to stop the spread of the infection.” Facebook users were free to comment under the post and to share it. Figure 1 shows a screenshot of the post in Polish. Facebook was chosen as a platform in particular because of the high number of Facebook users in Norway in general, and because this platform allowed us to target the position of users with high accuracy.

The campaign was disseminated using targeted Facebook Ads and was launched separately for each municipality or city district and each language. Target groups on Facebook were created by specifying the geographic position and language setting. It was not possible to launch a Facebook campaign when fewer than 100 users matched the targeting criteria. In cases where not enough users matched the language setting, target groups were formed on the basis of behavioral attributes such as “living abroad,” and interest segments specific to the users’ native country. Examples of the latter are as follows: “Syria national football team,” “BBC Urdu,” and “Turkish Kurdistan.” At the time of the trial, Somali and Tigrinya were not available as languages on Facebook. Users who spoke these languages were solely targeted on the basis of behavioral attributes. Lastly, the English campaign was disseminated on the basis of the attribute “living abroad” and the English user language setting.

The campaign was live for a 7-14–day period. The variation in timing was due to the time needed to roll out all the campaigns. Of the 117,436 participants in the intervention group, 52,565 lived in areas where it was possible to target the campaign in accordance with Facebook’s language settings, 58,336 lived in areas where it was only possible to target the campaign on the basis of behavioral attributes or interests, and 4700 lived in areas where it was not possible to launch the campaign, neither based on the participants’ background or language. We cannot know for certain to what extent the intervention group, living in areas where the campaign was rolled out in their language, was actually exposed to the intervention.

### Participants

The sample included 233,903 persons with a Norwegian national identity number, who are registered as a resident of a Norwegian municipality or city district (in Oslo, Bergen, and Trondheim), aged 18 years or older at the time of assignment, and registered are as having been born in Eritrea, Iraq, Pakistan, Poland, Russia, Somalia, Syria, or Turkey.

### Data Source and Outcomes

We used registry data from the emergency preparedness register for COVID-19 (Beredt C19), which was established by the NIPH in April 2020 [11]. The register contains data on all individuals in the Norwegian Population Register. Individuals were linked across data sources and over time, using an encrypted version of the unique personal identification number provided for every resident of Norway at birth or upon first immigration. In this study, we used individual data from the Norwegian Surveillance System for Communicable Diseases (MSIS) and the National Population Register. MSIS contains data on all polymerase chain reaction (PCR) and rapid tests taken in Norway at official test stations.

Data were also obtained from Facebook on whether it was possible to roll out the campaign, and on the reach, exposure, and frequency of the campaigns that were run in each cluster. These data were anonymized and used only in descriptive analyses, as data from Facebook cannot be linked directly to data on testing.

Data were aggregated into two time periods: baseline data included data collected 14 days to 1 day before the campaign commenced. Outcome data were measured from day 1 to day 14 and from day 1 to day 21 after the campaign started. Day zero was excluded from the analysis. The exclusion of day zero was prespecified in the protocol, and the rationale was that it was unlikely that the effects of the campaign could be observed on the first day.

The outcome was a dichotomous variable, measured at the individual level, indicating whether a person had taken a PCR or antigen test for SARS-CoV-2.

Time invariant covariates included country of birth, sex, and age. These variables were obtained from the National Population Register.

### Statistical Analysis

The coefficient of interest was *β*_1_ in regression analysis:

*y_j_*_[_*_i_*_]1_ = *α_j_*_[_*_i_*_]_ + *β*_1_*Treated_i_* + *β*_2_*y_i_*_0_ + ***X_i_****β*_3_ + *ε_j_*_[_*_i_*_]_*_t_*,

where *y_j_*_[_*_i_*_]1_ indicates whether the individual *i* in municipality or city district *j* has taken a COVID-19 PCR or rapid antigen test during the 2 weeks the campaign lasted. *y_i_*_0_ is the baseline value indicating whether the individual *i* has taken a COVID-19 test in the 2 weeks prior to campaign commencement. We adjust for the baseline value as is customary in randomized controlled trials (RCTs) [12,13]. ***X_i_*** is a vector of control variables.

As the outcome is binary, measured at the individual level, and the treatment was assigned at the cluster level, *j*, multilevel logistic regression analysis was performed to account for intraclass correlation.

The main specification does not take into account that it was not possible to administer the intervention in every municipality or city district assigned to the intervention group. As such, the results represent an “intention to treat” estimate.

### Sample Size Estimation

The sample size relied on the number of persons aged 18 years and older, born in Eritrea, Pakistan, Poland, Russia, Somalia, Syria, or Turkey, and residing in Norway. The number of languages and countries was determined by the availability of campaign material in the different languages, the distribution of these groups across Norway, and the size of the groups. The language groups were also selected on the grounds of a higher test positivity rate as opposed to the general population. Thus, our sample consisted of all eligible individuals, not a selected sample. Consequently, we performed no prior sample size estimation.

### Ethics and Privacy Issues

This study does not qualify as health research in the legal sense and hence does not need formal ethics approval. Participant consent was waived because this study was an evaluation of an intervention, using anonymous registry data already collected.

Personal data protection was ensured through the rigorous set up of the Beredt C19 where data on testing and country of birth were linked to the encrypted version of the unique personal identification number. Data from Facebook were collected and aggregated by Mindshare, and could not be linked directly to the registry data.

## Results

The campaign was live from June 1 to June 14, 2021. In 119 out of 189 municipalities or city districts, the campaign was rolled out on June 1, while in the remaining 70 municipalities, the campaign was live from June 6, 2021. In total, the campaigns reached more than 351,000 individual users. The users were likely exposed to the same campaign several times but in a different language, which was expected, as there were multiple languages per target country. The number of individual users far exceeds the 233,903 persons included in our target group, which likely entails that it also reached other users; for example, nonmigrant users, nonmigrants born to migrant parents, or migrants not born in 1 of the 8 target countries.

Summary statistics on the sample that was randomized are shown in Table 1 (Figure S1 in Multimedia Appendix 3 shows their localization on the map). The different migrants were quite evenly distributed between the intervention and control groups. In total, 42.7% of individuals in the intervention group and 42.2% of those in the control group were female. The average age was 41 years in the intervention group and 42 years in the control group. The average cluster sizes were similar in the intervention and control groups.

A striking difference between the groups is the preintervention COVID-19 fortnightly incidence rate, indicating that the randomization process did not yield a completely balanced sample. The number of fortnightly cases was much higher in the intervention group (123.6 cases per 100,000 population) than in the control group (79.6 cases per 100,000 population). This difference is likely to have influenced postoutcome testing, as the test-isolate-trace-quarantine strategy implies that a high number of persons will be tested in conjunction with COVID-19 outbreaks. In Figure S2 in Multimedia Appendix 3, we show that the higher number of cases is driven by a few observations, notably outbreaks in Hammerfest and Larvik municipalities. We therefore controlled for whether the individual had taken a COVID-19 test during the baseline period, and conducted a separate sensitivity analysis where we excluded the two pairs of clusters with the biggest preintervention differences in COVID-19 incidence.

**Figure 2:**
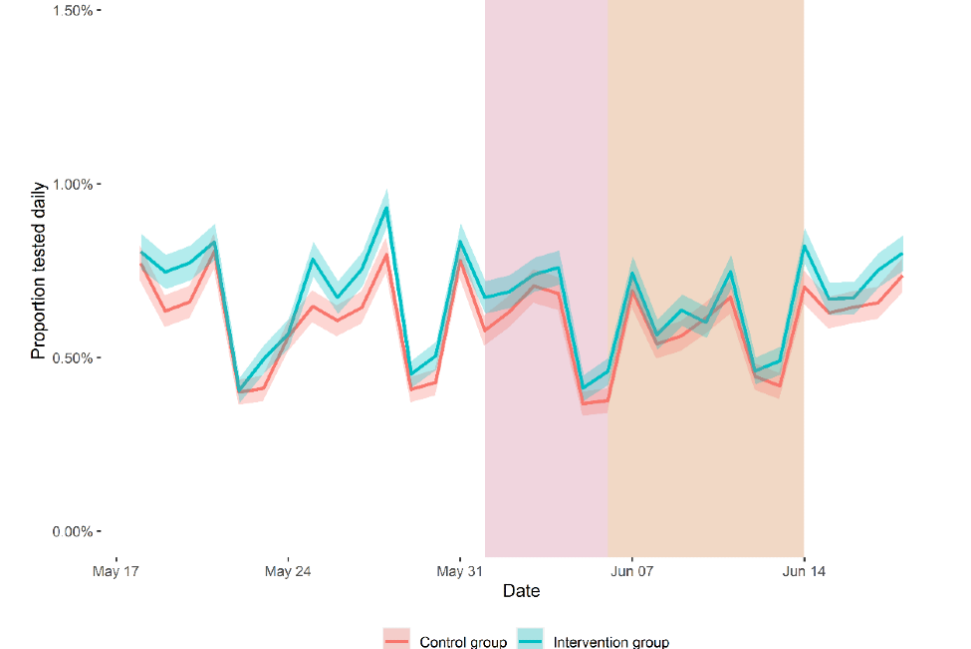
The daily proportion of the participants tested in the control and in the intervention groups. Error bars represent 95% CIs.

Figure 2 shows the proportion tested daily in the intervention and control groups. The proportion of individuals tested daily in the intervention group was higher than that in the control group on most days both before and after the intervention was carried out.

**Table 1:** Baseline demographic characteristics of participants and clusters by experimental arm (N=233,903).

Variable				Intervention group (n=117,436)		Control group (n=116,467)
**Individual-level characteristics**						
	**Gender, n (%)**					
		Female	50,194 (42.7)		49,176 (42.2)	
		Male	67,242 (57.3)		67,291 (57.8)	
	**Country of birth, n (%)**					
		Eritrea	9562 (8.1)		9662 (8.3)	
		Iraq	11,395 (9.7)		10,256 (8.8)	
		Pakistan	12,153 (10.3)		8387 (7.2)	
		Poland	45,319 (38.6)		49,247 (42.3)	
		Russia	8107 (6.9)		9204 (7.9)	
		Somalia	12,575 (10.7)		12,192 (10.5)	
		Syria	11,425 (9.7)		12,057 (10.4)	
		Turkey	6900 (5.9)		5462 (4.7)	
	Age, mean (SD)			41 (12)		40 (12)
**Cluster-level characteristics**						
	Clusters, n			191		191
	Cluster size, mean (SD; range)			609.8 (1154.5; 5-6931)		614.8 (1186.8; 2-7804)
	Population, n (mean)			2,858,940 (14,968.3)		2,649,940 (13,874.0)
	COVID-19 baseline fortnightly incidence (per 100.000)			79.6		123.6

Table 2 and Table S2 in the Multimedia Appendix 4 display the results of regression analysis. The odds ratio (OR) for the intervention group was 1.17 (95% CI 1.05-1.30; *P*=.004); that is, the campaign had a significant positive effect on the likelihood of undergoing a COVID-19 test. Whether a person had taken a COVID-19 test during the baseline period was a strong predictor of whether that person would take a test during the follow-up period (OR 5.34, 95% CI 5.12-5.56; *P*=.001). Controlling for age and sex did not affect the effect estimate.

**Figure 3:**
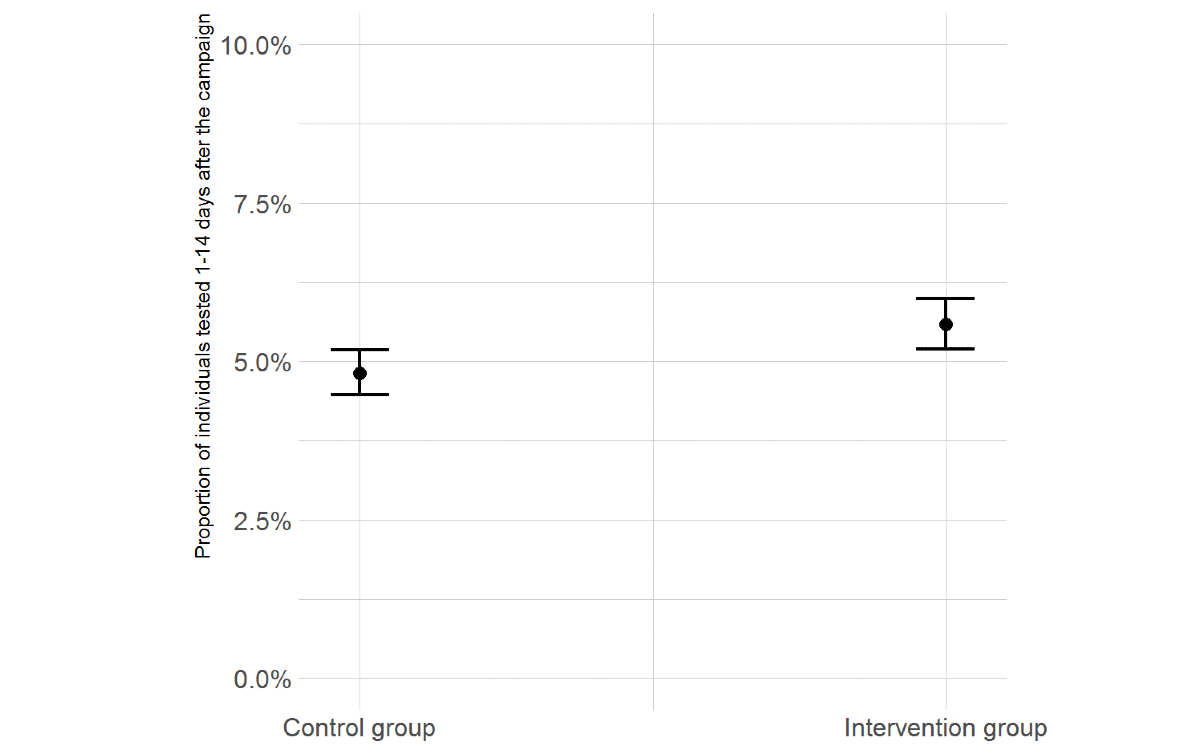
Predicted probabilities of conducting a COVID-19 test in the 14-day follow-up period. Error bars represent 95% CIs.

Figure 3 shows the predicted probability of undergoing a COVID-19 test in the 14-day follow-up period—this was 4.82% (95% CI 4.47%-5.18%) in the control group (areas without exposure to the campaign) and 5.58% (95% CI 5.20%-5.99%) in the intervention group (areas with exposure to the campaign). The 0.76 percentage point difference between the control and intervention groups converts to a 15.7% relative increase in test rates attributed to the campaign.

Table S1 in the Multimedia Appendix 3 shows the effects of the campaign when the follow-up period was 1-21 days after the campaign commenced rather than 1-14 days considered for our main analysis. The effect on testing remained significant (OR 1.13, 95% CI 1.02-1.25; *P*=.02). The results are also robust to the exclusion of the two pairs with the largest preintervention (baseline) differences in COVID-19 incidence. When we examined whether the effects differed in accordance with which targeting criteria was used to segment target groups, the results show that targeting based on the users’ language settings yielded a larger effect (OR 1.29, 95% CI 1.16-1.44; *P*<.001) than that based on behavioral attributes or interest (OR 1.11, 95% CI 1.00-1.24; *P*=.045). Not surprisingly, there was no significant difference in testing rates between individuals in the control group and those in areas where it was not possible to launch the campaign (OR 0.99, 95% CI 0.83-1.18; *P*=.89).

**Table 2:** Results of regression analysis.

Predictors			Tested (1-14 days)^a^				Tested (1-14 days)^b^		
			Odds ratio (95% CI)		*P* value		Odds ratio (95% CI)		*P* value
Intercept			0.04 (0.04-0.05)		<.001		0.04 (0.03-0.04)		<.001
Intervention group			1.17 (1.05-1.30)		.004		1.17 (1.05-1.30)		.004
Tested precampaign			5.34 (5.12-5.56)		<.001		5.24 (5.03-5.46)		<.001
Female			N/A^c^		N/A		1.25 (1.21-1.30)		<.001
**Age (years)**									
	25-39	N/A		N/A		1.13 (1.06-1.21)		<.001	
	40-44	N/A		N/A		1.12 (1.04-1.21)		.003	
	45-49	N/A		N/A		1.18 (1.09-1.27)		<.001	
	50-59	N/A		N/A		1.11 (1.03-1.19)		.007	
	60-70	N/A		N/A		1.02 (0.93-1.11)		.68	
	>70	N/A		N/A		0.65(0.56-0.76)		<.001	

^a^Model adjusted only for baseline value.

^b^Model adjusted for baseline value, gender, and age.

^c^N/A: not applicable.

## Discussion

### Principal Findings

We conducted this trial to assess the effect of a targeted social media campaign on the rates of testing among Norwegian residents born in Eritrea, Iraq, Pakistan, Poland, Russia, Somalia, Syria, or Turkey. We found a 15.7% relative increase in the proportion taking a COVID-19 test among those who were exposed to the campaign, or an absolute increase of 0.76 percentage points. A distinctive feature of this trial, that strengthens the significance of our findings, was that we were able to assess the effects on actual behavior and not only on intentions or beliefs [14].

We found that targeting in accordance with the users’ language setting yielded a larger effect than that based on behavioral attributes or interest. The content and message remained the same for both targeting approaches. A probable explanation is that the targeting based on users’ language settings reached a higher share of the participants than that based on behavioral attributes or interests.

### Comparison With Prior Work

We are aware of some trials with a similar objective, assessing the effects of social media campaigns on COVID-19–related preventive behavior, COVID-19 knowledge, and intent toward COVID-19 prevention.

We identified 3 RCTs that test the effect of social media campaigns on COVID-19 prevention knowledge. Alsan et al [15] examined how messages read by physicians who varied in age, gender, race, and ethnicity influenced knowledge, beliefs, and practices related to COVID-19. Physician-delivered messages increased knowledge of COVID-19 symptoms and prevention methods for Black and Latinx respondents. The results illustrate that tailoring the message to the targeted groups may increase the effectiveness of the interventions. Similarly, Vandormael et al [16] have conducted a web-based RCT to investigate the effect of a short video on improving COVID-19 prevention knowledge and behavioral intent toward COVID-19 prevention. They concluded the following: “Short, wordless, animated videos, distributed by health authorities via social media, may be an effective pathway for rapid global health communication during health crises” [16]. Lastly, the results of Alatas et al [17] suggest that celebrity endorsement in a social media campaign in Indonesia influenced beliefs about vaccination and knowledge of immunization.

Only Breza et al [18] investigated how social media advertisements affect actual COVID-19 preventive behavior. They showed that short messages recorded by health professionals before the winter holidays in the United States and sent as advertisements to social media users led to reductions in travel (–0.993 percentage points in high-intensity counties, *P*=.002) and a decrease in COVID-19 infection at the zip code level in the 2-week period starting 5 days post holiday (3.5%, *P*=.01) [18].

Our results are aligned with those of the aforementioned studies. We demonstrated that targeted advertisements on social media with short, simplified messages can be a valuable tool in the authorities’ toolbox in times of a pandemic. Targeted advertisements can reach audiences that are not easily reached by traditional modes of communication. Furthermore, conveying the message in the mother tongue of the audience can increase trust in the message, although we did not test this specifically.

The effect size was small, which was not unexpected, considering that the COVID-19 prevalence was quite low in the weeks before campaign onset, and that the intervention was a part of a longer ongoing campaign. Norway saw a large increase in test rates among migrants toward the end of 2020 and in early 2021, after the “Get tested” campaign was launched and amid media attention on the high incidence of COVID-19 among migrant groups [19]. It is therefore likely that toward the end of 2021, the target groups were experiencing a certain advertisement fatigue, and that the main message of the campaign was well known both in the control group and the intervention group.

### Limitations

There are some limitations associated with the findings of this study. First, the design of the trial limited the scope and content of the campaign, and the likely external validity. Because we needed to roll out separate campaigns in all areas, it was too arduous to also put out videos or other types of campaign material. In a real-life setting, it would not be necessary to limit users to a very specific geographic unit, the costs of administrating the campaign would be lower, and there would be room for other modes of communication.

Another limitation was that it was not possible to identify the exact effect of actually seeing the posts on testing behavior, as data on reach could not be directly linked with registry data on testing. We know that 82% of the population aged 18 years or older has a Facebook profile [7], but this is only one of many social media platforms. Nevertheless, the total number of users speaking different languages, who were reached, suggests that a large share of the target group was exposed to the campaign.

A related limitation is that the campaign may have had spillover effects on the control group; for example, persons in the intervention group may have shared the post to their Facebook friends and followers. Such organic reach could not be measured, nor was it avoidable. Similarly, the message could have spread from the intervention group to the control group by word of mouth. Spillover effects may have led to an underestimation of the true effect.

Future research could look into effects of communication campaigns on other types of COVID-19 preventive behavior. We also suggest that future campaigns consider using the emergency broadcast capability inherent in cell phone services to deliver targeted advertisements.

### Conclusions

Seeing a native-language post on Facebook, in a clear language easily understood manner regardless of health literacy, explaining that testing is simple and can be taken at no cost rendered our target group more likely to take a COVID-19 test. This study demonstrated that targeted social media advertisements sponsored by health authorities can influence individual behavior in an infection control–friendly direction during a pandemic.

## Appendix

Multimedia Appendix 1 Protocol: Evaluation of social media campaign to increase COVID-19 testing in migrant groups: A cluster randomised trial.

Multimedia Appendix 2 CONSORT extension for Cluster Trials.

Multimedia Appendix 3 Additional results.

Multimedia Appendix 4 Findings of the random effects model.

## Abbreviations

Beredt C19: emergency preparedness register for COVID-19
MSIS: Norwegian Surveillance System for Communicable Diseases
NIPH: Norwegian Institute of Public Health
OR: odds ratio
PCR: polymerase chain reaction
RCT: randomized controlled trial

## References

[1] Labberton A, Godøy A, Elgersma I, Strand BH, Telle K, Arnesen T, Nygård KM, Indseth T (2022). SARS-CoV-2 infections and hospitalisations among immigrants in Norway-significance of occupation, household crowding, education, household income and medical risk: a nationwide register study. Scand J Public Health.

[2] Indseth T, Grøsland M, Arnesen T, Skyrud K, Kløvstad H, Lamprini V, Telle K, Kjøllesdal M (2021). COVID-19 among immigrants in Norway, notified infections, related hospitalizations and associated mortality: A register-based study. Scand J Public Health.

[3] Berg SH, O'Hara JK, Shortt MT, Thune H, Brønnick KK, Lungu DA, Røislien J, Wiig S (2021). Health authorities' health risk communication with the public during pandemics: a rapid scoping review. BMC Public Health.

[4] Brekke J (2021). Informing hard-to-reach immigrant groups about COVID-19—Reaching the Somali population in Oslo. J Refug Stud.

[5] Weekly reports for coronavirus and COVID-19. Norwegian Institute of Public Health.

[6] Vinjerui KH, Elgersma IH, Fretheim A (2021). Increased COVID-19 Testing Rates Following Combined Door-to-Door and Mobile Testing Facility Campaigns in Oslo, Norway, a Difference-in-Difference Analysis. Int J Environ Res Public Health.

[7] Ipsos SoMe-tracker Q2?21 er endelig tilgjengelig. Ipsos.

[8] Create a blocked randomisation list. Sealed Envelope.

[9] So J, Kim S, Cohen H (2016). Message fatigue: Conceptual definition, operationalization, and correlates. Communication Monographs.

[10] Kim S, So J (2018). How Message Fatigue toward Health Messages Leads to Ineffective Persuasive Outcomes: Examining the Mediating Roles of Reactance and Inattention. J Health Commun.

[11] Emergency preparedness register for COVID-19 (Beredt C19). Norwegian Institute of Public Health.

[12] (2018). Different ways to estimate treatment effects in randomised controlled trials. Contemp Clin Trials Commun.

[13] Barnett AG, van der Pols JC, Dobson AJ (2005). Regression to the mean: what it is and how to deal with it. Int J Epidemiol.

[14] Potter W (2012). Media Effects.

[15] Alsan M, Stanford FC, Banerjee A, Breza E, Chandrasekhar AG, Eichmeyer S, Goldsmith-Pinkham P, Ogbu-Nwobodo L, Olken BA, Torres C, Sankar A, Vautrey P, Duflo E (2021). Comparison of Knowledge and Information-Seeking Behavior After General COVID-19 Public Health Messages and Messages Tailored for Black and Latinx Communities : A Randomized Controlled Trial. Ann Intern Med.

[16] Vandormael A, Adam M, Greuel M, Gates J, Favaretti C, Hachaturyan V, Bärnighausen T (2021). The Effect of a Wordless, Animated, Social Media Video Intervention on COVID-19 Prevention: Online Randomized Controlled Trial. JMIR Public Health Surveill.

[17] Alatas V, Chandrasekhar A, Mobius M, Olken B, Paladines C (2019). When Celebrities Speak: A Nationwide Twitter Experiment Promoting Vaccination In Indonesia. National Bureau of Economic Research.

[18] Breza E, Stanford FC, Alsan M, Alsan B, Banerjee A, Chandrasekhar AG, Eichmeyer S, Glushko T, Goldsmith-Pinkham P, Holland K, Hoppe E, Karnani M, Liegl S, Loisel T, Ogbu-Nwobodo L, Olken BA, Torres C, Vautrey P, Warner ET, Wootton S, Duflo E (2021). Effects of a large-scale social media advertising campaign on holiday travel and COVID-19 infections: a cluster randomized controlled trial. Nat Med.

[19] Indseth T, Godøy A, Kjøllesdal M Covid-19 etter fødeland fra mars 2020 til februar 2021. Folkehelseinstituttet.

