# Supplementary material for: The Evaluation of a Social Media Campaign to Increase COVID-19 Testing in Migrant Groups: Cluster Randomized Trial
Source: J Med Internet Res. 2022 Mar 24;24(3):e34544. doi: 10.2196/34544 (PMC8955230; doi:10.2196/34544)
Supplement: Multimedia Appendix 1 [file jmir_v24i3e34544_app1.docx]

Protocol: Evaluation of social media campaign to increase COVID-19 testing in migrant groups: A cluster randomised trial

# Administrative information

This protocol was based on the SPIRIT 2013 Checklist.

## Title

Evaluation of social media campaign to increase COVID-19 testing in migrant groups: A cluster randomised trial

## Trial registration

## Protocol version

Version 1, 14.06.2021

## Funding

Funded by Norwegian Institute of Public Health and Norwegian Directorate of Health

## Roles and responsibilities

Primary (lead) investigator: Ingeborg Hess Elgersma, [ingeborghess.elgersma@fhi.no](mailto:ingeborghess.elgersma@fhi.no), +47 91584785

Atle Fretheim, [Atle.Fretheim@fhi.no](mailto:Atle.Fretheim@fhi.no)

Christine Engh Hansen, Mindshare, [christine.hansen@mindshareworld.com](mailto:christine.hansen@mindshareworld.com)

Anita Thorolvsen Munch, [Anita.Thorolvsen.Munch@helsedir.no](mailto:Anita.Thorolvsen.Munch@helsedir.no)

Thor Indseth, [Thor.Indseth@fhi.no](mailto:Thor.Indseth@fhi.no)

# Introduction

## Background and rationale

Many migrant groups are disproportionally affected by Covid-19. Consequentially, health authorities have carried out several media campaigns targeting migrants. The campaigns are traditionally measured by the reach and impressions the campaigns generate, in addition to pre- and post-testing messages in terms of how the campaigns affect knowledge and confidence. However, the end rationale of these specific campaigns – namely behavioural change - is not adequately tested and documented. This study aims at closing this gap by assessing the effects of a media campaign targeting migrants in Norway. The proposed campaign aims at increasing the test rate among immigrants.

Technological advances render it possible to successfully target social media campaigns to persons living within prespecified geographical areas. By randomizing which areas are exposed to the campaign, it is possible to assert the effects of the campaign on the share of conducted covid-19 tests among migrants. The campaign is distributed according to the migrant’s mother tongue.

## Objectives

The objective of the study is to evaluate the effect of the social media campaign on the rate of migrants testing for Covid-19. Furthermore, the study will differentiate between migrant groups to investigate whether the effect varies between groups.

## Trial design

The trial is designed as a cluster randomised trial. The clusters were block randomized (block size two), according to size of each cluster, i.e. the number of migrants born in the countries of interest residing in each cluster.

The RCT constitutes a superiority parallel group trial design. The clusters were allocated either to the intervention or to the control group. The clusters were split evenly between treatments. After randomisation, the participants will stay in their assigned treatment for the duration of the study.

The framework of the trial is to test whether there is a significant difference in the testing rate between those targeted with the social media campaign and those who were not.

# Methods: Participants, interventions, and outcomes

Study setting

The study is a population-wide, registry-based study. The participants are randomized into treatments without their knowledge. The data is obtained from the Emergency preparedness register for COVID-19 (Beredt C19) and is collected only in Norway. A list of treated and untreated clusters will be made available concurrent to the publishing of a report of the findings from the study.

Eligibility criteria

The trial includes all persons with a Norwegian national identity number, registered as resident of a Norwegian municipality or city district (in Oslo, Bergen and Trondheim), of age 18 or older and registered as born in either Eritrea, Pakistan, Poland, Russia, Somalia, Syria or Turkey.

Intervention

The intervention is a 14-day media campaign targeting persons living in Norway born in the 7 countries mentioned above. The campaign is distributed on social media in Tigrinya, Polish, Urdu, Somali, Russian, Kurmanji, English, Arabic and Sorani.

By applying a Facebook and Instagram segmentation, targeting and positioning approach, the campaign should reach those in the intervention group but not in the control group. The message of the campaign is on the importance of testing for covid-19, and that getting tested for the corona virus is easy and free of charge.

The adherence will be monitored by the campaign team via Facebook’s overview. As an integrated part of social media is its sharing function, it is not possible to fully avoid that the campaign may be shared with the people in the control groups.

By applying a broad segmentation approach so to reach as many people in the treatment group as possible, it is also possible that the campaign reaches the non-immigrant population, or those immigrants out of the scope of this trial.

## Outcomes

The primary outcome is the increase in the test rate the campaign yields. The proportion tested will be calculated by dividing the number of individuals who have taken a test between the 1^st^ and 14^th^ day after the campaign commenced on the total number persons in the group.

To account for baseline bias, we suggest using the test rate the 14 days prior to the start of the intervention as the baseline. Day 0, the start of the campaign, is not included in the baseline measurement nor the treatment measurement. We will also assess the impact of the campaign for the 21-day period following the campaign launch.

## Timeline

| 14 days prior | Start of the campaign (day 0) |  | 14 days after start |
| --- | --- | --- | --- |
|  |  |  |  |

## Sample size

The sample size relies on the number of persons of age born in Eritrea, Pakistan, Poland, Russia, Somalia, Syria and Turkey residing in Norway. Per 19^th^ of May 2021 the sample compromised 234 112 individuals from 386 clusters (municipalities or city districts). The number of languages/countries were determined based on the availability of campaign material in the different languages, the spread of these groups across Norway and the size of the groups. These language groups were also selected on the grounds of a higher rate of positive tests as opposed to the general population ^1^ Thus, our sample will consist of all eligible individuals, not a selected sample.

# Methods: Assignment of interventions

The assignment of the intervention was conducted as follows:

Team member 1 (Ingeborg Hess Elgersma) created a list of clusters (municipalities and city districts) based on the total number of individuals eligible to take part in the trial. The data stems from the Norwegian Institute of Public Health. The list was sorted (stratified) based on the cluster size (the total number of individuals in each cluster).

Team member 2 (Atle Fretheim) created a list of pairwise random orders of pairs of A and B, using the randomisation software provided by [www.sealedenvelope.com](http://www.sealedenvelope.com) (<https://www.sealedenvelope.com/simple-randomiser/v1/lists>), for block randomisation with block size of two.

Team member 3 (Thor Indseth) put these two lists together and returned the list to team member 1. It was decided prior to this procedure that group A was to be assigned the intervention and that group B was assigned the control state.

Team member 1 created a list of only those clusters assigned to the intervention. This list was forwarded to the provider of the campaign.

The trial participants were blinded to their assignment to the interventions.

# Methods: Data collection, management, and analysis

## Data collection methods

The effects of the campaign will be assessed by utilizing register-based data. The data stems from the Norwegian Emergency preparedness register for COVID-19 (Beredt C19). The register contains data from, among other things, the National Population Register and the Norwegian Surveillance System for Communicable Diseases (MSIS). The National Population Register is used to identify the age, place of residence and background of the sample population. The data from MSIS is applied to identify the number and time of tests taken by any individual. Both PCR and antigen tests will be counted towards the total number of tests.

In addition, data from Facebook will be used to describe the reach of the campaign, e.g. the number of views, the number of clicks, the number of times the content was shared and the discussions and comments generated by the posts. Data will be collected by Mindshare, the contractor that assists with the targeting on Facebook and Instagram.

## Data management

Data is stored in a secure sever in the data infrastructure of the Norwegian Institute of Public Health.

## Statistical methods

As the outcome is binary, measured on the individual level, and the treatment is assigned on the cluster level, multilevel analysis will be performed to account for intra class correlation in the data.^2^ The exact model is yet to be specified, but will account for the reduced effective sample size due to the data clustering.

In addition to the main analysis, a subgroup analysis will be conducted letting the effect of the campaign vary between groups (Somalis, Pakistanis etc.).

# Methods: Monitoring

## Data monitoring

As this is not a clinical trial, a data monitoring committee (DMC) is not relevant.

## Harms

As this kind of targeting is already a part of authorities’ strategy towards informing immigrant groups, no adverse events or other unintended effects are expected.

## Auditing

No auditing is planned.

# Ethics and dissemination

## Research ethics approval

Research ethics committee/institutional review board (REC/IRB) approval for the study is not required under Norwegian law, as it does not classify as health research.

## Protocol amendments

Any amendments to the protocol will be communicated in the final journal article and report.

## Consent or assent

Consent is not required under Norwegian law.

## Confidentiality

As this is a registry based RCT, we do not store personal information about the participants elsewhere than as described under data management and data collection methods.

## Declaration of interests

Christine Engh Hansen is an employee at Mindshare, a marketing company that sells social media-related services

## Access to data

The access to the dataset will remain limited to the researchers at the Norwegian Institute of Public Health. The team will strive to produce a synthetic dataset, if not too arduous. Scripts will be made available upon requests.

## Ancillary and post-trial care

No provisions

## Dissemination policy

The results of the trial will be communicated in a report. The aim is also to report the study in an academic journal. The statistical code will be made available upon request.

# Appendices

## References

1. Indseth T, Godøy A, Kjøllesdal M, et al. *Covid-19 etter fødeland fra mars 2020 til februar 2021*. Folkehelseinstituttet, 11 March 2021.

2. Kim H-Y, Preisser J, Rozier R, et al. Multi-level analysis of group-randomized trials (GRT) with binary outcomes. *Community Dent Oral Epidemiol* 2006; 34: 241–51.
