# Supplementary material for: The Evaluation of a Social Media Campaign to Increase COVID-19 Testing in Migrant Groups: Cluster Randomized Trial
Source: J Med Internet Res. 2022 Mar 24;24(3):e34544. doi: 10.2196/34544 (PMC8955230; doi:10.2196/34544)
Supplement: Multimedia Appendix 3 [file jmir_v24i3e34544_app3.docx]

# Additional results


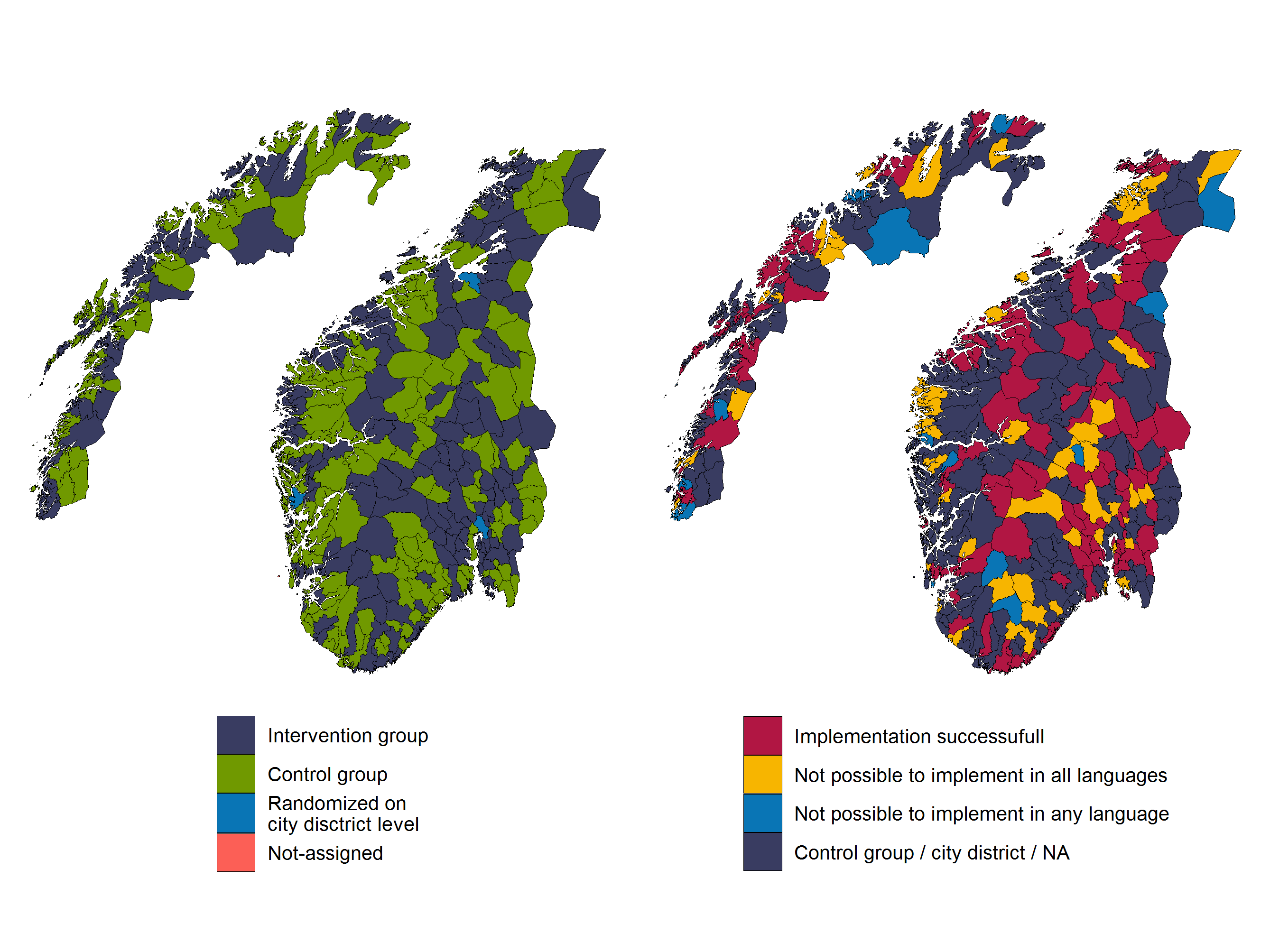


Figure S1: Implementation status

Table S1: Additional regression results

|  | **Tested(1-21 days)** | | **Tested(1-14 days)** | | **Tested(1-14 days)** | | |
| --- | --- | --- | --- | --- | --- | --- | --- |
| *Predictors* | *OR (P)* | *CI* | *OR (P)* | *CI* | *N* | *OR (p)* | *CI* |
| (Intercept) | 0.07 (**<.001)** | 0.06 – 0.07 | 0.04 (**<.001)** | 0.04 – 0.05 |  | 0.05 (**<.001)** | 0.04 – 0.05 |
| Intervention  group | 1.13(**.02)** | 1.02 – 1.25 | 1.18 (**.003)** | 1.06 – 1.31 |  |  |  |
| Implementation status (ref: control group) |  |  |  |  | 118 302 |  |  |
| Not implemented |  |  |  |  | 4700 | 0.99 (.890) | 0.83 – 1.18 |
| Implemented by interests |  |  |  |  | 58 336 | 1.11 (**.045**) | 1.00 – 1.24 |
| Implemented by language setting |  |  |  |  | 52 565 | 1.29 (**<.001)** | 1.16 – 1.44 |
| Tested  pre-campaign | 4.30 (**<.001)** | 4.14 – 4.47 | 5.43 | 5.21 – 5.66 (**<.001)** |  | 5.33 (**<.001**) | 5.11 – 5.55 |
| σ^2^ | 3.29 | | 3.29 | | 3.29 | | |
| τ_00_ | 0.15 _municipality-district_ | | 0.16 _municipality-district_ | | 0.15 _municipality-district_ | | |
| ICC | 0.04 | | 0.05 | | 0.04 | | |
| N | 382 _municipality-district_ | | 378 _municipality-district_ | | 382 _municipality-district_ | | |
| Observations | 233903 | | 231933 | | 231933 | | |
| Marginal R2 / Conditional R2 | 0.041 / 0.081 | | 0.053 / 0.097 | | 0.054 / 0.096 | | |


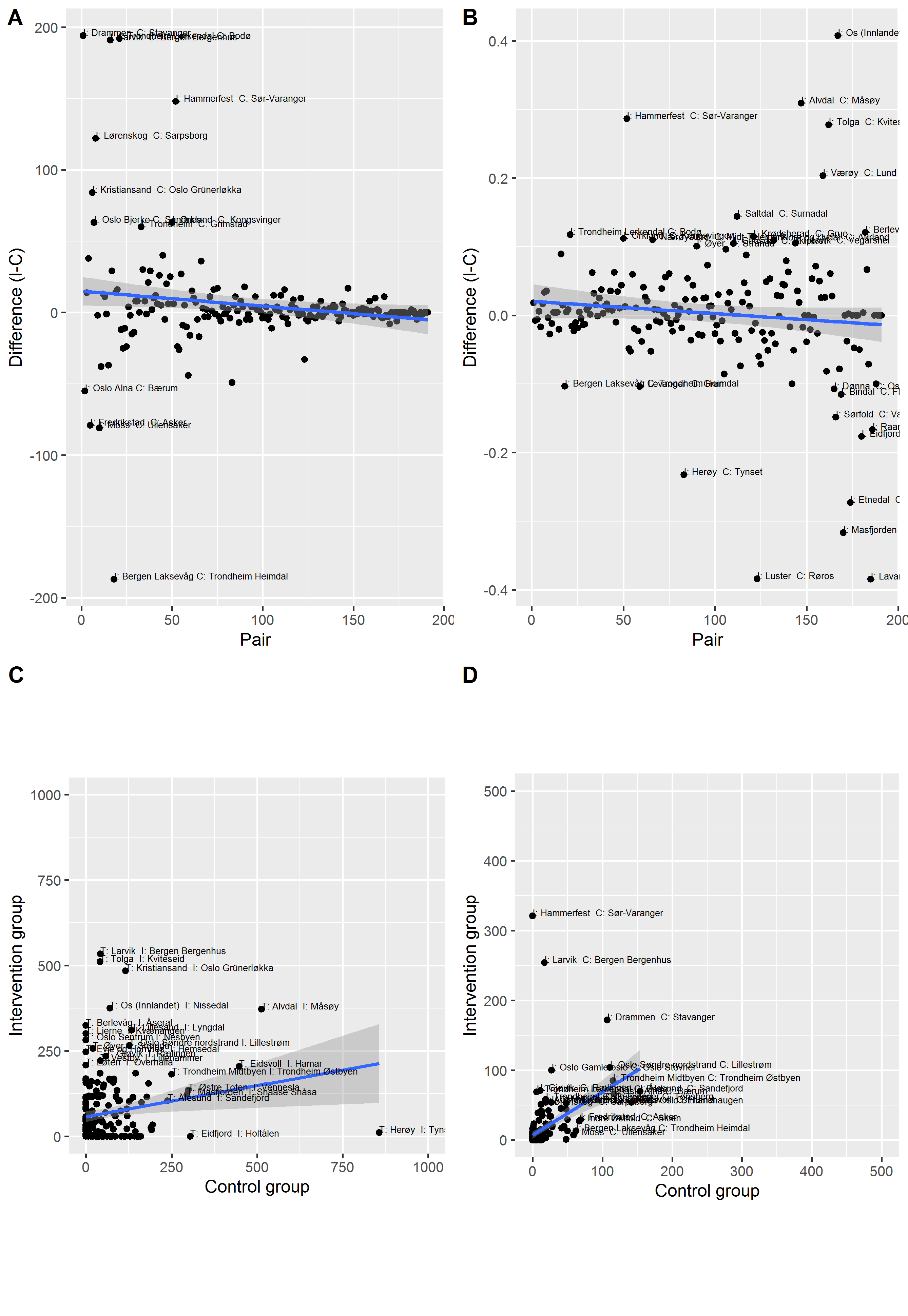


Figure S2: Panel A: Pairwise absolute pre-campaign difference in the number of tested in each municipality / city district. Panel B: Pairwise relative pre-campaign difference in the number of tested in each municipality / city district. Panel C: Difference between pairs in COVID-19 Baseline Fortnightly Cases per 100 000. Panel D: Difference between pairs in the number of COVID-19 Baseline Fortnightly Cases.
