# Supplementary material for: The Evaluation of a Social Media Campaign to Increase COVID-19 Testing in Migrant Groups: Cluster Randomized Trial
Source: J Med Internet Res. 2022 Mar 24;24(3):e34544. doi: 10.2196/34544 (PMC8955230; doi:10.2196/34544)
Supplement: Multimedia Appendix 4 [file jmir_v24i3e34544_app4.docx]

*Table S2****:*** *Findings of the random effects model.*

| Parameter | Tested (1-14 days)^a^ | Tested (1-14 days)^b^ |
| --- | --- | --- |
| Population variance (σ^2^) | 3.29 | 3.29 |
| Between-group variance (τ_00_; municipality-district) | 0.16 | 0.16 |
| Intraclass correlation coefficient | 0.05 | 0.05 |
| Individuals (municipality-district), n | 382 | 382 |
| Observations, n | 233,903 | 233,903 |
| Marginal *R*^2^/conditional *R*^2^ | 0.053/0.096 | 0.059/0.102 |

^a^ Model adjusted only for baseline value.

^b^ Model adjusted for baseline value, gender, and age.
